# Continuous sedation until death: the everyday moral reasoning of physicians, nurses and family caregivers in the UK, The Netherlands and Belgium

**DOI:** 10.1186/1472-6939-15-14

**Published:** 2014-02-20

**Authors:** Kasper Raus, Jayne Brown, Clive Seale, Judith AC Rietjens, Rien Janssens, Sophie Bruinsma, Freddy Mortier, Sheila Payne, Sigrid Sterckx

**Affiliations:** 1 Department of Philosophy and Moral Sciences, Ghent University, Blandijnberg 2, Ghent, Belgium; 2 End of Life Care Research Group, Ghent University and Vrije Universiteit Brussel, Laarbeeklaan 103, Brussels, Belgium; 3 School of Nursing and Midwifery, De Monfort University, The Gateway, Leicester, UK; 4 Department of Sociology and Communications, Brunel University, Uxbridge, Middlesex, UK; 5 Department of Public Health, Erasmus MC, Dr. Molewaterplein 50, Rotterdam, The Netherlands; 6 Department of Medical Humanities, VU Medical Centre, Van der Boechorststraat 7, Amsterdam, The Netherlands; 7 International Observatory on End of Life Care, Lancaster University, Furness College, Lancaster, UK

## Abstract

**Background:**

Continuous sedation is increasingly used as a way to relieve symptoms at the end of life. Current research indicates that some physicians, nurses, and relatives involved in this practice experience emotional and/or moral distress. This study aims to provide insight into what may influence how professional and/or family carers cope with such distress.

**Methods:**

This study is an international qualitative interview study involving interviews with physicians, nurses, and relatives of deceased patients in the UK, The Netherlands and Belgium (the UNBIASED study) about a case of continuous sedation at the end of life they were recently involved in. All interviews were transcribed verbatim and analysed by staying close to the data using open coding. Next, codes were combined into larger themes and categories of codes resulting in a four point scheme that captured all of the data. Finally, our findings were compared with others and explored in relation to theories in ethics and sociology.

**Results:**

The participants’ responses can be captured as different dimensions of ‘closeness’, i.e. the degree to which one feels connected or ‘close’ to a certain decision or event. We distinguished four types of ‘closeness’, namely *emotional, physical, decisional*, and *causal*. Using these four dimensions of ‘closeness’ it became possible to describe how physicians, nurses, and relatives experience their involvement in cases of continuous sedation until death. More specifically, it shined a light on the everyday moral reasoning employed by care providers and relatives in the context of continuous sedation, and how this affected the emotional impact of being involved in sedation, as well as the perception of their own moral responsibility.

**Conclusion:**

Findings from this study demonstrate that various factors are reported to influence the degree of closeness to continuous sedation (and thus the extent to which carers feel morally responsible), and that some of these factors help care providers and relatives to distinguish continuous sedation from euthanasia.

## Background

Providing sedation in end-of-life care involves lowering or removing consciousness so that a patient no longer experiences distressing symptoms, whose relief may be judged impossible by other means. This practice is known as sedation until death, palliative sedation, or terminal sedation, and can be given intermittently or continuously until the patient’s death. In this paper we focus on continuous sedation until death (hereinafter CS).

Several guidelines concerning the practice have been published [1-5]. However, CS also remains subject to considerable clinical, legal and ethical debate [6,7]. One contested issue is the difference between CS and euthanasia. Some commentators argue that CS causes death and often amounts to ‘slow euthanasia’ [8-10]. Others argue that even if CS does not hasten death, it causes patients to permanently lose the capacity to communicate, and may thus amount to an imposition of social death on the patient [11,12]. However, other commentators, as well as many guidelines [1-5], maintain that there is a distinct difference between CS and euthanasia. Distinguishing the two arguably is one of the main purposes of the Dutch national guideline on sedation [5,12], a guideline that has served as the basis on which other sedation guidelines have built.

Furthermore, some relatives and health care professionals (here collectively referred to as care providers) involved in CS experience moral and/or emotional distress. While some insight has been provided into the nature of this distress [13-16], relatively little is known about what may influence carers’ ability to cope with such distress. In this paper we focus on the language used by care providers when contemplating cases of CS in which they have been involved.

This paper is based on the UNBIASED study (UK Netherlands Belgium International Sedation Study), conducted in the UK, Belgium and The Netherlands. The specific research questions for this paper are:

1) How do physicians, nurses, and relatives report dealing with the emotional impact of being involved in continuous sedation until death?

2) How is this linked to their understanding of their own moral responsibility?

These research questions will be answered by schematising the data using a four point scheme in which we will identify four types of ‘closeness’, by which we mean the degree to which one feels connected with or responsible for certain decisions or events^a^.

Although our study includes data from three different countries, our research questions do not focus specifically on national differences. For each quote used in this paper we specify whether the respondent was Belgian, Dutch or British, but our focus is on the everyday moral reasoning that was common to participants of the three countries.

## Methods

The protocol for this study has been published elsewhere [17] and contains a detailed report of the methods used, so only a summary is provided here. The UNBIASED study is a qualitative study undertaken in the UK, The Netherlands, and Belgium, involving interviews with physicians, nurses, and relatives who had recently been involved in the care for a sedated patient or loved one, asking them about their experiences. The study was approved by the responsible Institutional Review Boards (IRB’s) in all participating countries.

Interviews were conducted with 57 physicians (17 UK; 22 NL; 18 BE), 73 nurses (25UK; 28 NL; 20 BE), and 34 relatives (8 UK; 13 NL; 13 BE) who were involved in a total of 84 cases of CS (22 UK; 35 NL; 27 BE). As some physicians and nurses were interviewed about more than one case and more than one relative was present for some of the interviews with relatives, this makes for a total of 82 interviews with physicians, 78 interviews with nurses and 32 interviews with relatives. Interviews were undertaken between January 2011 and May 2012. As we were unable due to issue of confidentiality to access clinical notes the physicians who had agreed to participate in our study were asked to contact us if they had been involved in caring for a patient older than 18 years of age, diagnosed with cancer and to whom sedating medications had been administered continuously until death with the aim of reducing difficult or refractory symptoms. Cases of both light and deep continuous sedation were included (although the great majority of cases given to us by physicians were cases of *deep* continuous sedation). To avoid recall bias, physicians were interviewed as soon as possible. Participating physicians were also asked to identify the nurse most involved in each case and they were subsequently invited to take part in the study and, where they agreed, promptly interviewed. We also asked the health care professionals interviewed to identify the most involved relatives in each case who were then invited to take part in an interview for the study via the physician. Thus, ideally, for each single case of continuous sedation at the end of life, an interview was conducted with the physician, nurse and relative who were most involved in this case.

For maximum variety we included cases of deceased patients who had received CS either while at home, in a hospital setting (mostly oncology wards) or in a specialist palliative care setting (hospices for The Netherlands and the UK, and palliative care units attached to hospitals for Belgium). Care homes for the elderly were not included as these services are organised very differently in the UK, The Netherlands and Belgium, making comparison difficult.

Potential interviewees received an information sheet about the study and were given the opportunity to ask questions. Following this a consent sheet was signed and participants were reminded that withdrawal from the study was possible at any time. A short questionnaire providing demographic information was also completed. The interviews themselves were semi-structured, guided by an *aide-memoire* containing general questions and prompts (See Additional file 1 and Additional file 2). The focus of each interview was the participants’ experiences with, and perceptions of, their involvement in the care of a patient who had received CS until death. Questions were also asked about participants’ general views and attitudes towards continuous sedation until death.

Interviews were anonymised and transcribed verbatim. The Dutch and Belgian interviews were translated into English by professional translators. Important quotes and passages were double-checked by the researchers for language and interpretation. The data was analysed thematically applying a constant comparative method [18] using NVIVO™ to allow the results to emerge directly from the data. Initially open coding involved text segments being given a descriptive code, followed by the development of a larger coding tree which combined codes into larger themes or categories. Coding was done by researcher KR and checked independently by SS and CS. The combining of codes into larger categories resulted in the four-point scheme reported in the results section. Differences in coding or analysis were always discussed and researchers were always able to reach consensus. Finally, our findings were compared with others and explored in relation to theories in ethics and sociology.

This process from open coding to combining codes into larger categories resulted in a four point scheme which we report in this article^b^. Though it of course concerns a schematic way of organizing a complex data set, we believe it captures the data. As we did not start with a preconceived theory, the result is a new scheme, although we will argue that it fits in well with other findings in ethics and sociology.

## Results

Many participants in our study discussed the personal impact of being involved in CS until death. In explaining what they thought influenced this impact we found that many commonly referred to the degree of ‘closeness’ (our term) between their own actions and what happened [19]. ‘Closeness’ is an important moral feature, as the degree of closeness one experiences can influence one’s experience of moral distress or the degree to which one feels morally responsible for certain actions or events. Our analysis of the data revealed that closeness, as it was reported by the respondents, had four dimensions – *decisional*, *causal*, *physical* and *emotional* closeness – each of which will be discussed below. Besides affecting the emotional impact of CS on respondents, some dimensions helped respondents distinguish CS from euthanasia. The distinction between CS and euthanasia lies at the heart of moral dilemmas experienced by some care providers and is a particular concern in the Dutch national guideline [5,12].

### Emotional and physical closeness

Respondents made it clear that being involved in continuously sedating a patient until death had great emotional impact and that this was influenced by the degree to which they felt *emotionally* and *physically* close to the patient receiving CS.

Regarding *emotional* closeness, as may be expected, relatives reported a high level of emotional involvement and nurses reported being more emotionally involved than physicians who were less immersed in the daily care of patients. Emotional closeness was greatest when participants developed a personal bond, or identified with the patient. Conversely, emotional closeness was resisted by, for example, stressing differences between themselves and the patient. For example, a Dutch physician said:

Ah yes, Mrs. X, somebody about my age, I am a year younger. It is very challenging to admit such a young woman. She had a child of 16; my oldest is 16. I identified myself greatly with her. But those are the only similarities, because she was divorced, had a bad marriage and was always dependent on her mother.

(Dutch physician on a palliative care ward, case 25)

Respondents could also be seen to reduce emotional closeness by stressing the need to stay ‘professional’, as several physicians and nurses put it. For example, a Belgian nurse explained that, for her, caring for dying patients is particularly difficult:

Especially when they are also young, then it is particularly difficult. We are only human after all… But on the other hand, what I think doesn’t really matter then. At that moment, you need to be professional. It’s what he wants and not what I would [want].

(Belgian nurse on oncology ward, case 18)

Or as explained by a Dutch physician:

Grief can be so palpable and feel like a blanket. To enter such a room and think “ooh I have to wiggle through this, I have to stay professional, I have to make sure I remain standing”, and just take two breaths.

(Dutch physician on a palliative care ward, case 25)

*Physical* closeness also influenced emotional impact, something which mostly affected relatives and nurses (who provided the majority of the physical care). Some care providers found that being physically close to a sedated patient made it easier for them to cope when sedation was successful in making the patient comfortable. However, in cases where patients still seemed to be suffering, being physically close to the patient could be distressing.^c^ For example, a Dutch relative said regarding a situation where physicians and nurses were unable to get the patient calm:

***Relative****: He was… his body really stayed restless, shocking… A fight, really fighting.*

***Interviewer****: And how was it for you to be there?*

***Relative****: Terrible…*

(Dutch relative in hospice, case 23)

A relative of a UK patient described being highly distressed by watching her husband in his last days, sedated but displaying symptoms such as excessive phlegm:

I mean the last couple of days, there was all this gargling and everything going on, were pretty horrific, you know. And I mean I was told that he was on so much stuff that he wouldn’t have known about it, although when they suctioned him out he’d still got, like, a gagging reflex and everything. But that was awful because I could do nothing for him … (weeping) and that wasn’t nice to see him like that.

(UK relative in hospice, case L2)

Though not the same, *emotional* and *physical* closeness were sometimes seen as related, as being physically close to a patient often resulted in feeling emotionally close to that patient. For example, a Dutch nurse commented on a case she had encountered earlier in her career where a baby was admitted to the hospice, and reported being grateful she was not on duty then, because she knew that by being physically involved, she would develop an emotional bond which would have made the baby’s death difficult for her. She said:

I was very glad that I wasn’t involved in the care at that moment, so that I couldn’t build a relationship.

(Dutch nurse in hospice, case 23)

### Decisional closeness

Deciding to use CS is a major decision as it involves reducing a patient’s consciousness, often to a level at which the patient is no longer able to communicate, until death. Being closely involved in such a decision can have a profound emotional impact and this also influenced interviewees’ perceptions of their own moral responsibility.

The interviewees most often described factors which decreased decisional closeness. There was a major difference between physicians, nurses, and relatives in their decisional authority^d^. Nurses often reported not having final responsibility for the decisions surrounding CS and often saw their role as more ‘advisory’, reducing their decisional closeness. A Dutch nurse, for example, described being involved in the sedation process without having final responsibility:

So the physician does take the decision [to start CS], but we of course also watch how the patient is doing and whether it is time [to start] or not.

(Dutch Nurse on oncology ward, case 9)

Relatives sometimes also experienced a lesser role in decision making, as this UK relative reported:

***Interviewer:*** *were people talking to you about the decision to increase the amount of sedation in the syringe driver, and who, if anybody, did talk to you about that?*

***Relative:*** *Erm… I… I can’t actually put my finger on that one. The only thing I can say is that [a particular carer] was very, erm, keeping me informed and everything else. Erm, I mean the decision really wasn’t down to me, it was down to, down to the medical sort of people…*

(UK Relative, case L2)

While in these instances decisional authority rested with the physician or the medical team, in other cases respondents described the authority for the decision as resting with the patient. In all three countries there were examples of nurses and physicians stressing that the decision to initiate or increase sedation was *not* in fact theirs, but the patient’s. Thus, a Belgian nurse said regarding a decision to initiate CS:

It is not my choice and that is… then you can cope and then you are, ethically speaking, OK with it. (…) And if you keep that in mind, I think …this may sound very silly, but I want to compare it with [when] someone in your ward wants to change sex, that you must not judge them. Whether you have difficulty with that or not, it’s the patient’s choice… And you must show that you give them the best possible care because that is your job as a professional.

(Belgian nurse on oncology ward, case 18)

A UK nurse commented in a similar vein when asked how the use of CS made her feel:

Make me feel? That that’s what he wanted. I think, if that’s what people want, then why not? Each to their own, isn’t it?

(UK nurse in home care setting, case L2)

Decisional closeness for the individual was also reduced by depicting the decision as a team decision, so that responsibility was shared. A statement by a UK physician illustrated this, also showing that a ‘team’ might involve almost everyone involved in the case. When asked whether shared decision-making made the decision easier, the physician replied:

Yes…it’s always a collaborative decision-making in some respect. The… final sort of, erm… responsibility probably lies with me…but in fact the actual decision’s been made with nursing input…and in fact the patients and staff and the relatives’ wishes as well.

(UK physician in home care setting, case GPROT1)

Decisional closeness was also reduced by emphasising the fact that no other decision could have been taken, thus reducing participants’ scope for exercising agency. In these cases, the situation is conferred with a degree of inevitability whereby, given a patient’s physiological state and medical history, the only option was CS. Thus a Dutch physician said:

I think there was no other option for this lady. She was really…this was a lady who really suffered from the situation and for whom we really did not have another option besides this. So I think [sedation] was, medically speaking, inevitable.

(Dutch physician on oncology ward, case O3)

Similarly, for those who were aware of the existence of protocols or guidelines and used these^e^, decisional closeness and therefore personal agency could be reduced by stating one was only following guidelines (e.g. the Dutch national guideline). For example, a UK physician talked about drafting a guideline for sedation for treatment-resistant agitation (one of the most common indications for CS), to make care providers more comfortable:

We are going to draw up some guidelines for people with resistant agitation, so that when somebody is this bad, we’ll feel more comfortable and we’ll say, ‘No, this is what we need to do’ rather than (…) worry about how you’re gonna do it.

(UK physician in hospice, case L74)

### Causal closeness

As mentioned above, decisional closeness refers to the degree in which respondents felt ‘close’ to the decision that was taken. However, interviewees also discussed their perceived closeness to the causal chain of events in administering sedative drugs (and thereby reducing or taking away the patient’s consciousness) and in the eventual death of the patient. We refer to this as ‘causal closeness’. Like decisional closeness, causal closeness affected respondents’ perceptions of moral responsibility.

It is often recommended that physicians act in a way that would increase their causal role in sedation over that of nurses, for example by always being present when sedation is started up, or by administering the drugs themselves rather than asking nurses to do this [5]. In practice however the difference between physicians and nurses was sometimes minimal as many nurses described being actively involved in administering sedative drugs at various points in the process, including, in some cases, initiation. Thus, whereas there were differences between nurses and physicians with regard to emotional and physical closeness, these differences were less noticeable with regard to causal closeness.

However, there were circumstances where care providers experienced an uncomfortable sense of having been too causally close. In such situations, they often felt distressed, as illustrated, for example, by the experience recounted by a Belgian nurse regarding a case where she had to inject a patient with intravenous Midazolam:

Once I gave someone intravenous Midazolam, and I thought, I’m injecting this person to death. And that really isn’t a pleasant feeling. I felt like, I’m pushing her under water, still, still, still, still, still, still, still, still, still, still, and now you may come back up. That’s the feeling I had and I don’t want to have it again.

(Belgian Nurse on palliative care ward, case P23)

An important way in which the sense of causal closeness was reduced was by adopting the notion that administering CS allowed the natural disease process to take its course. Describing CS in this way displaced causal responsibility for the patient’s death from the carer onto the natural order of events. A Belgian physician commented:

Um, yes, of course in palliative sedation (…) it is not the intention to make people die (…). The intention is that we make them comfortable (…) and then let nature run its course.

(Belgian physician on oncology ward, case O14)

Many respondents pointed out that intention was what distinguished a death following CS from a death following euthanasia. While euthanasia was reported as involving a deliberate action aimed at ending a patient’s life, in CS potential life-shortening can be seen as a side-effect and is thus less causally close. Examples were plentiful, but one of the most interesting comments came from a Dutch nurse, whose comments show how reducing her sense of causal closeness had a major impact on her own feelings about the procedure:

***Interviewer****: In your opinion, is there a very big difference between palliative sedation and euthanasia?*

***Nurse****: Yes… I think euthanasia is really clear… yes you clearly inject somebody away. And yes just dead. And with sedation you take away a person's consciousness, but not that person’s life. Nature, or let me put it this way, the natural process, can go on despite the sedation…*

***Interviewer****: And that makes a big difference for you?*

***Nurse****: That makes a huge difference for me… We also do euthanasia on the ward and I myself cannot take part in it, because I would have a huge guilt feeling.*

(Dutch nurse on oncology ward, case O33)

Another example is that of a Belgian nurse who described the difference between euthanasia and CS:

What is euthanasia? That’s an injection, that’s immediate, but sedation is then actually to let nature run its course.

(Belgian nurse on palliative care ward, case P5)

The view that CS does not shorten life is also something that involved a reduction in causal closeness by asserting that the respondents’ actions did not actively contribute to a patient’s death. For example, when a Dutch physician was asked whether she found it difficult to intervene in the dying process, she replied:

Well … sedation of course is not a way to speed up the dying process, it is something meant to lighten the burden of life. And as far as that goes, I have no problem with it. I myself have never experienced euthanasia, but I personally would find that much more difficult because that is truly intervening to shorten life. That would affect me a lot more [than continuous sedation].

(Dutch physician on oncology ward, case 41)

Another element influencing causal closeness was the time between the start of CS and the death of the patient. Once CS has been initiated the patient can stay alive for several days (or even weeks), making it less clear whether, and to what extent, the participants actions directly caused the patient’s death. Accordingly, some participants reported distress in cases where patients died very shortly after having received CS, as the following statement of a UK nurse demonstrates:

If I was to give, I don’t know, 20mgs midazolam it might have been what he needed, and then he died 20 minutes later…what does that say to the family? How do you justify that? In a Court of Law would that be accepted? Do you see what I mean?

(UK nurse on oncology ward, case O2)

In a second example, a UK nurse talked about cases where patients die shortly after the start of CS, which she found difficult:

Especially when it’s so quick… because quite often you think… that is dreadful, absolutely dreadful, when they go soon after the injection.

(UK nurse in Hospice, case L3)

### Stressing benefits over harms

As has been shown, many care providers described situations in which their decisional and emotional closeness was lowered, and how this influenced the emotional impact of their involvement and their perceptions of moral responsibility.

However, closeness is just one factor that was felt to influence the emotional impact of involvement in CS. Respondents who experienced closeness did not always say this was associated with emotional distress. Many participants stated that the benefits outweighed the harms of sedation. Employing this type of ‘balancing’ reasoning was another way for participants to cope with their feelings of moral responsibility, particularly in cases in which participants felt emotionally, physically, decisionally, or causally close to the delivery of CS. Sometimes, if they were convinced that in a particular case the benefits significantly outweighed the harm, respondents would even assert that decisional and causal closeness was desirable as long as one is acting from motives of compassion. A Belgian nurse, for example, stressed that she felt very privileged to have been able to play a part in making a decision that contributed significantly to reducing a patient’s suffering.

***Nurse****: I was able to contribute something so that he no longer had to suffer. So at that moment you take… you take part in an important medical decision that can be of great help.*

***Interviewer****: Yes, and that in turn helps you to deal with it?*

***Nurse****: Of course.*

(Belgian nurse on oncology ward, case O18)

This nurse clearly felt the weight of this ‘important medical decision’, but dealt with this heavy sense of responsibility, derived from her decisional closeness, by stressing the overwhelming benefit to the patient.

The idea that, by providing CS, they were helping a suffering person achieve the best experience of the end of life that was possible was an important element in dealing with moral and emotional distress. A Belgian physician described having issues with CS, but being able to cope with his involvement because he knew that it was of great help in easing patients’ suffering, saying:

I can reconcile myself with that. I don’t lie awake because I know that I am helping people with it, um, but I continue to find it difficult.

(Belgian physician on oncology ward, case O14).

Some respondents expressed the fear that CS has a potential to shorten life, and that therefore in using it, one may be causally responsible for hastening a patient’s death. Stressing the benefits of CS sometimes helped care providers deal with this perceived causal closeness. When asked whether she believed continuous sedation shortened life in a certain case, a Dutch nurse answered:

That is a difficult one, very difficult. I do not know. Yes maybe it was just the little push that this gentleman needed, that is possible. But I granted him that. And yes… yes… yes I have no problem with that, then I have something like okay, why not? Why not sedate someone then?

(Dutch nurse in hospice, case P24)

## Discussion

It is clear that many physicians, nurses and relatives were emotionally affected by their involvement in CS. The emotional impact of being involved in a case of CS was highest when respondents felt physically and emotionally close to the sedated patient. This concurs with available research [13-16]. The management of emotions such as anger, fear, grief and distress in both intimate and public contexts, including health care contexts, is a long established field for sociological research. Notably, for example, Hochschild’s [20] concept of ‘emotional labour’ (i.e. the deliberate regulation of one’s own and one’s clients emotions as part of one’s job) [20] has been applied in studies of the caring professions [21-23].

However, the emotional element of care work is not solely confined to emotional labour. As a way of coping with the emotions aroused by such significant actions as providing CS, care providers may want to mitigate the degree to which they feel morally responsible. Certain factors can influence what we have called the *closeness* of a carer to a particular decision or practice.

Regarding physical and causal closeness, care providers mostly used language that appeared to reduce the closeness between themselves and the decision to use CS. In this respect, our results match well with Bandura’s [19] well known work in describing certain ways in which people are able to disconnect their actions from events, thereby reducing their sense of moral responsibility, understood as the degree to which they feel they can be blamed or praised for their role in those events [19]. Bandura [19] talks of ‘moral disengagement’ resulting from such decreases in closeness to particular actions [19]. This ‘moral disengagement’ is not necessarily rare or sinister, but is rather a mechanism to help people cope with difficult decisions in everyday life. The specific mechanisms of ‘moral disengagement’ described by Bandura include describing one’s actions in euphemistic ways (as for example in saying that CS allows the process of dying to occur naturally), by displacing responsibility (for example to the patient or the natural order), and diffusing responsibility (for example, saying that CS was a joint team decision, or the product of following guidelines). These ways of helping people cope with their own feelings of moral responsibility, were all evident in the interviews.

Thus in CS there are many ways in which care providers can influence their experience of emotional, decisional, and causal closeness, perhaps then leaving more ‘wiggle room’ [9] for decision making. For many respondents, this possibility of ‘wiggle room’ in CS seemed to distinguish the practice from euthanasia where, at least for the physician, certain dimensions of closeness are much more difficult to deny or reduce (although some who invoke a far-reaching concept of self-determination by the patient would perhaps displace responsibility to the patient entirely). Injecting a lethal dose of medication is clearly the cause of a patient’s death and, as provided by the euthanasia laws in Belgium, The Netherlands, and Luxembourg, the physician has final authority over the decision. Since respondents reported decisional and causal closeness as being emotionally and morally difficult, the possibility of a more easily reduced closeness in CS might be an attractive aspect of that practice.

Unsurprisingly, as both Belgium and The Netherlands have legalised euthanasia, there was more discussion of this topic in the Belgian and Dutch interviews. The acceptance of shortening life as a way to relieve suffering in some cases might partly explain why Belgian and Dutch physicians, nurses, and relatives were seemingly less troubled by the possibility of CS shortening life. UK physicians and nurses on the other hand were more concerned about this, as well as being concerned not to give the impression, in the eyes of other caregivers or family members, that CS had shortened life.

Distancing oneself as a way of coping with involvement in a difficult practice might be perfectly understandable at times, and may sometimes even be beneficial (e.g. when it results in truly shared decision-making). However, reducing closeness may not always be ethically desirable. For example, the view that death following CS is natural, could be considered in several respects to represent a distortion of the truth. Raus et al. [24], who suggest five characteristics that are commonly seen as key elements of a ‘natural’ death (- deep sleep, fading away, internal causes, no prolonging or shortening of life, and no agency -) to the case of CS until death, and who argue, in line with e.g. Seymour et al. [25] and Billings and Block [8], that the resemblance between death following CS and a ‘natural’ death are merely a mimicry or a simulation.

Furthermore, although shared decision-making is generally considered to be the best model for end-of-life decision-making, it is questionable whether the displacement or diffusion of responsibility is always desirable. Sharing a decision can be done for the wrong reasons, for example in order to not have to assume responsibility. Also, placing *all* responsibility on the patient might be distressing for the patient (if they are aware of it) while reducing the role of the carers to the role of ‘executors’ of the patient’s wishes.

This paper reports data from the UNBIASED study which, a qualitative study conducted with the same methodology in three different countries. Quantitative studies have already shown that major differences exist regarding the decision-making and performance of CS in The Netherlands, Belgium and the UK [26,27]. This study provides valuable insights into the commonalities between physicians, nurses, and relatives from these three countries in how they described describe and use continuous sedation until death.

This study also has some limitations. For ethical reasons cases were reported via physicians, creating a possible bias in the types of cases that were put forward for inclusion in the study. This was countered by giving physicians clear criteria and asking them to report every case that fitted those criteria. Next, this study included only adult cancer patients to guarantee a sufficiently similar sample for Belgium, The Netherlands and the UK, and may therefore not be generalizable to CS in patients suffering from diseases other than cancer. However, in response to general questions physicians *did* discuss continuous sedation for patients suffering from other diseases, and no major differences were reported. Given its design, the authors do not see the findings as being generalizable to all physicians, nurses and relatives involved in CS in the three countries, however it is hoped that the arguments resonate with the experience of others and that the findings are transferable. Moreover, there were fewer hospital cases in the UK, whereas in Belgium comparatively fewer cases came from a specialist palliative care setting. However, we have succeeded at including experiences of many different types of sedation from the three settings (home care setting, hospital setting, and specialist palliative care setting), to obtain maximal variety in our sample. Finally, we did not explore in detail the impact of country or clinical setting on physicians’, nurses’, and relatives’ attitudes towards CS at the end of life. Such comparisons will be the subject of later papers arising from this project.

Our study also has implications for policy. Despite the fact that many guidelines stress that CS is ‘normal medical practice’, for many physicians, nurses, and relatives the initiation of CS can be an emotionally distressing decision. Policy-makers should be attentive to this, for example by allowing physicians or nurses the chance to discuss their distress afterwards or giving nurses the possibility to be less involved in some cases (i.e. when there is a great risk of developing a strong personal tie with the patient). Using our findings, a case can also be made for more education for physicians and nurses centred on decision making in relation to their perception of moral responsibility, and, for example, for including relatives with experience of the impact of CS in such educational projects.

## Conclusion

This paper provides a discussion of the emotional and moral impact on caregivers of providing continuous sedation until death. Our interviews have highlighted factors influencing emotional involvement as reported by care providers. We have attempted to show how these different factors can be understood as variations in ‘closeness’ in its different dimensions: emotional, physical, decisional and causal. Finally, we have argued that our study shows how perceptions of these different dimensions of closeness play an important role in care providers’ understanding of their own moral responsibilities. This gives an important insight into participants’ reasoning when involved in a far reaching practice such as continuous sedation at the end of life.

## Endnotes

^a^‘Proximity’ could be used as a synonym for ‘closeness’ in the sense used in this paper.

^b^Note that in reporting our results we omit absolute numbers of how often and in how many different interviews a certain dimension of closeness was mentioned or alluded to. The issue of whether or not one should give numbers or percentages in qualitative research is a topic of debate. We agree with Pope et al. [28] that ‘qualitative research does not seek to quantify data’ ([28] p. 114), and we feel that the number of times a certain dimension of closeness was mentioned is far from always a good measure of the importance of that dimension. For our method of analysis we have focused heavily on the emphasis people put on dimensions of closeness during the interviews and how this related to other parts of their story rather than how many times an aspect of closeness was mentioned.

^c^See Bruinsma et al. [14] for a detailed account of relatives’ experiences with continuous sedation.

^d^‘Decisional authority’ in this context should not be interpreted in a legalistic sense. It refers to the role people played or perceived to have played in the decision-making process regarding the sedation.

^e^Abarshi & Payne (Abarshi E, Payne S: Awareness of the European Association for Palliative Care's (EAPC) Recommended Framework for the Use of Sedation in Palliative Care, forthcoming) conducted a study among members of the European Association of Palliative Care (EAPC) which showed that although many members were aware of the existence of guidelines (the EAPC guideline, a national guideline, or both), a significant number of respondents was not aware of any guidelines.

## Competing interests

The authors declare that they have no competing interests.

## Authors’ contribution

All authors were involved in drafting the UNBIASED research protocol and in executing the research. KR did the primary analysis and created the first draft. SS and CS were responsible for checking the coding and analysis. All authors commented on earlier drafts of the article and all authors read and approved the final manuscript.

## Pre-publication history

The pre-publication history for this paper can be accessed here:

http://www.biomedcentral.com/1472-6939/15/14/prepub

## Supplementary Material

Additional file 1 Aide memoire interviews with physicians and nurses, questions (bold) and subsidiary prompts. Click here for file

Additional file 2 Aide memoire interviews with informal care-givers, questions (bold) and subsidiary prompts. Click here for file
