# Supplementary material for: Continuous sedation until death: the everyday moral reasoning of physicians, nurses and family caregivers in the UK, The Netherlands and Belgium
Source: BMC Med Ethics. 2014 Feb 20;15:14. doi: 10.1186/1472-6939-15-14 (PMC3942295; doi:10.1186/1472-6939-15-14)
Supplement: Additional file 2 — Aide memoire interviews with informal care-givers, questions (bold) and subsidiary prompts. [file 1472-6939-15-14-S2.doc]

**Aide memoire interviews with informal care-givers, questions (bold) and subsidiary prompts**

**All Respondents - symptoms in the last weeks of life**

**Can you tell me what you remember about the care of X in the last week(s) of their life?**

*Did X have any symptoms or distress? If so how do you remember these being treated? Where there any problems in managing X’s symptoms? In what way were you involved in the care and decision making? Who else was involved?*

**Did anyone ever give them something to relax?**

**Section A - for those who recall use of sedation**

**What do you remember about how the decision to use sedation was made? (*note: researcher should use the same term used by the carer*)**

*Why was sedation used? Who was involved and what did they do? (Patient, relatives, nurses, doctors, others)*

**Before sedation therapy started, were you and X given information about it?**

*What were you told? Who talked to you? Did you understand what you told? Were you kept informed as time went on?*

**Can you tell me what you recall about the use of sedation for X and how you think they responded to the treatment?**

*Could they still hold a conversation? Were they asleep, some or all of the time? For how long was X sedated? Was this continuous or did they have periods when they were not sedated? What other sorts of treatment can you recall that they received? (e.g. artificial nutrition and hydration?) How effective were the drugs in relieving symptoms do you think?*

**Looking back now, how do you feel things went?**

*What makes you feel that? Could anything have been better? What difference do you think the sedation treatment made to X's death?*

**Section B – for those with no recollection of the use of sedation**

**What do you remember about how decisions in X’s treatment were made?** *What were the reasons for that treatment? Who was involved and what did they do? (Patient, relatives, nurses, doctors)?*

**Where you told about what was happening with X’s care?**

*What were you told? Who talked to you? Did you understand what you were told? Were you kept informed as time went on?*

**Looking back now, how do you feel things went?**

*What makes you feel that?* *Could anything have been better?*

**Is there anything else you would like to tell me?**
